# Feasibility Study of Scrap Grading Systems Based on Three-Dimensional Vision Technology

**DOI:** 10.3390/s26061792

**Published:** 2026-03-12

**Authors:** Guangda Bao, Wenzhi Xia, Yun Zhou, Zhiyou Liao, Ting Wu, Haichuan Wang

**Affiliations:** 1 School of Metallurgical Engineering, Anhui University of Technology, Ma’anshan 243032, China; bgd98@ahut.edu.cn (G.B.); xwz423628@163.com (W.X.); zhouyun@ahut.edu.cn (Y.Z.); lzhy@ahut.edu.cn (Z.L.); 2 Key Laboratory of Metallurgical Emission Reduction & Resource Recycling (Ministry of Education), Anhui University of Technology, Ma’anshan 243002, China

**Keywords:** scrap grading, 3D vision technology, point cloud segmentation, multi-view reconstruction

## Abstract

To address the inefficiency and unfairness of traditional manual scrap sorting, we propose the application of 3D vision technology for grading in this work. The multi-view 3D reconstruction algorithm achieves an accuracy within 1 mm in both synthetic and real scrap scenes. This level of accuracy meets the requirements for scrap grading. Subsequently, an automated processing workflow in a non-overlapping scrap scenario is investigated, in which a pipeline based on the multi-view reconstruction integrating point cloud segmentation technique is proposed. Four-point cloud clustering segmentation methods, including Euclidean clustering, Kmeans, DBSCAN and Region Grow, are compared, and it is found that the Euclidean-clustering-based point cloud segmentation algorithm provides the best overall trade-off, achieving an mIoU score of 99.35%, while the thickness measurement error is less than 0.5 mm. The workflow suggests improved robustness and reliability compared to using a single 2D image for thickness inference. These results indicate that 3D vision may provide a valuable basis for the future development of scrap grading systems.

## 1. Introduction

With an annual crude steel output of 1 billion tons in China, the resulting scrap demand of approximately 300 million tons underpins a scrap supply chain with an annual economic scale of nearly USD 100 billion. As the sole large-scale green substitute for iron ore, scrap demand is poised to grow [1,2], driven by the steel industry’s green transition. The sector is constrained by two key issues: the lack of a unified grading system in the supply chain, resulting in pricing difficulties, and the complex nature of scrap sources coupled with a reliance on inefficient manual sorting due to automated equipment shortages. This leads to inconsistent scrap quality, severely affecting smelting stability.

**Figure 1:**
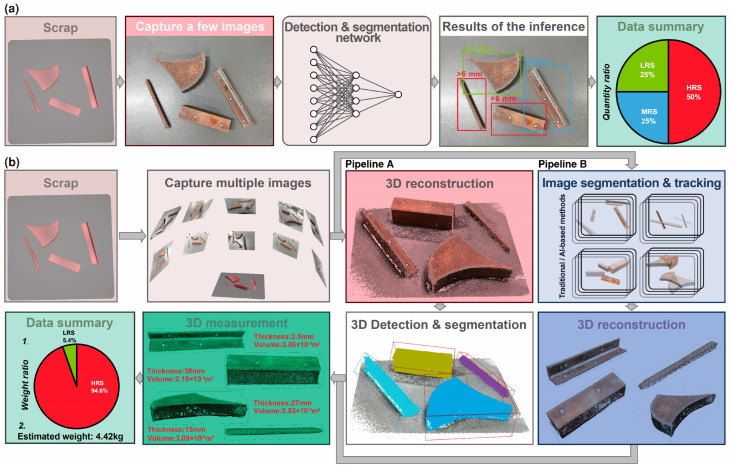
Architectural comparison of the scrap grading frameworks: (**a**) mainstream 2D workflow, (**b**) proposed 3D workflow.

According to the Chinese National Standard GB/T 39733-2024 [3], the thickness of scrap metal serves as the primary criterion for its grading. The specific grading criteria are detailed in Table 1. Currently, most steel companies still primarily rely on visual inspection and simple sampling measurement by relevant personnel for scrap grading and pricing, which leads to fairness issues and can easily breed corruption. An intelligent grading system for scrap is illustrated in Figure 1a, outlining the overall 2D framework. Based on 2D image recognition, scrap is divided into ‘<3 mm’, ‘3–6 mm’, ‘>6 mm’, ‘Galvanized’, ‘Greasy dirt’, ‘Paint’, ‘Inclusion’ and other categories [2,4,5]. Qiu [6] and Duan [7] employed the Yolov3 algorithm to achieve target detection in scrap images, implementing modifications to the Yolo network structure to enhance the detection accuracy. By proposing a variety of deep learning algorithms that integrate different attention mechanisms, Xiao [4,5] established a notable advantage in accuracy when compared to traditional manual quality assessment methods. Based on insights shared by on-site workers and the author’s practical investigation and analysis, the current 2D recognition approach faces the following issues. (i) The aforementioned deep learning approach primarily achieves recognition through the acquisition of texture and shape information about the object in question [8]. There is no significant correlation between the scrap thickness and its shape and texture, namely, the thickness of scrap with different shapes and textures may vary randomly. (ii) Scrap thickness is often several orders of magnitude different relative to length and width dimensions, and the information exposed in the image is usually insufficient for the algorithm to learn the thickness dimensions. (iii) The identification of scrap with varying thicknesses (‘<3 mm’, ‘3–6 mm’, ‘>6 mm’) constitutes a fine-grained identification challenge [9], whereas the current coarse-grained identification approach fails to yield optimal recognition outcomes. (iv) The system employs a single-view detection method, a technique that is inherently susceptible to errors and exhibits suboptimal robustness. In other words, if the same batch of scrap is rolled and naturally slid, the position and posture of the collected scrap will change. In some cases, the thickness direction is not exposed, resulting in a significant discrepancy between the two detections of the same scrap batch. (v) Value assessment based on the quantity ratio of scrap categories frequently leads to inaccuracies, as it ignores weight differentials. In conclusion, the veracity of acquiring 3D scrap information, particularly in regard to thickness, based on 2D images is questionable. This type of system is merely a transitional solution to the issue of scrap grading.

Building upon 2D visual recognition frameworks, this study proposes a novel 3D vision scheme for scrap thickness identification. The adopted 3D vision technology has found widespread application in numerous fields [10,11], with autonomous driving [12] being a prominent and rapidly advancing example. The proposed scheme integrates modules for 3D reconstruction, object recognition, and post-processing, as depicted in Figure 1b. In contrast to conventional 2D methods (Figure 1a), the presented 3D framework processes multi-view images to reconstruct comprehensive geometric attributes, such as thickness distribution and volumetric parameters, thereby significantly improving the robustness of grading accuracy and cost-efficiency analysis. The framework adopts a dual-pipeline architecture distinguished by their recognition mechanisms: Pipeline A identifies objects through iterative point cloud feature extraction and spatial pattern matching, while Pipeline B accomplishes recognition by aggregating multi-view image features and establishing 2D–3D correspondence mapping. This study focuses specifically on Pipeline A, a point-cloud-centered approach that employs geometric feature analysis to extract 3D spatial patterns, thereby establishing a dimensionally distinct alternative to image-based 2D paradigms in industrial settings. This study reconstructs scrap scenarios using photogrammetric methods and establishes a dedicated 3D dataset to evaluate the reconstruction performance. Furthermore, point cloud processing and segmentation experiments are conducted on the reconstructed data to validate the effectiveness of various algorithms in controlled scrap scenarios.

In this paper, Section 2 describes the establishment details of a multi-view scrap dataset. The construction process of the multi-view reconstruction algorithm and the methods related to point cloud processing are given in Section 3. Section 4 describes the evaluation criteria of the algorithm, analyzes the effect of the multi-view reconstruction algorithm in the scrap scene, and finally proposes an automated pipeline for scrap grading in an unoccluded and non-overlapping scene. Section 5 and Section 6 outline the intended industrial applications as well as a summary of the paper and a discussion of future work.

## 2. Materials

After a comprehensive search, it was found that publicly available datasets for scrap steel are extremely scarce. The only dataset identified in the literature is a segmentation dataset [13], which merely provides top-view images of truck containers transporting scrap steel rather than multi-view images. Moreover, there is almost no existing research on multi-view 3D reconstruction for scrap steel.

### 2.1. Multi-View Dataset of Single Scrap

**Figure 2:**
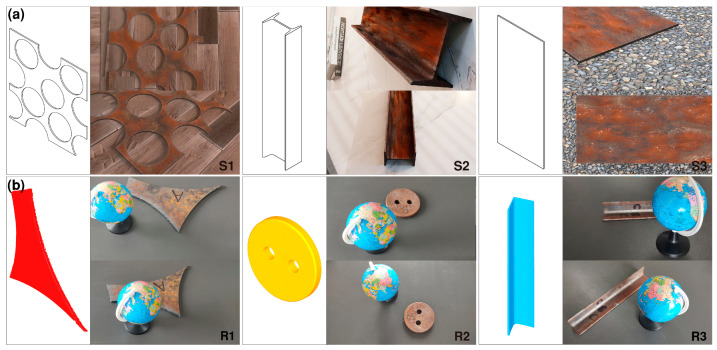
Multi-view scrap dataset: (**a**) synthetic and (**b**) real.

Characteristics of high controllability and diversity and low labeling and synthesis costs make synthetic data widely used in the field of CV research, especially in the field of 3D vision, whereas it is very expensive to produce real datasets. To ensure the fidelity of the scene, the scrap CAD models were created in Blender, and the materials and environmental lighting of the ground and other objects were set based on the blenderkit plugin. A virtual camera was used to take photos around the scrap. The rendering engine was selected as Cycles, the image size was 1920 × 1080 pixels, and the maximum sampling value of rendering was 2000 to reduce the noise in the rendered image and improve the image quality. Blender (V2.8) incorporates an embedded Python interpreter, enabling control of the multi-view image rendering process through either custom Python scripts or the BlenderProc [14] library. In the synthesis work, real-scale scrap models were used. For example, the CAD model for scrap group S2 was based on a 10# hot-rolled I-beam. Therefore, the CAD model was used as GT for accuracy evaluation. The CAD model of the synthetic dataset and some dataset images are shown in Figure 2a.

Although the fidelity of synthetic datasets is quite good, real-world collected scrap images have richer details, and the environment during the real-world collection process is complex, with issues such as noise, images being out-of-focus, and underexposure. This makes the reconstruction of real-world datasets more difficult but closer to the industrial field usage environment. Similar to the synthetic datasets, real scrap from a steel plant with characteristics of ship dismantling scrap, prompt industrial scrap, and construction scrap were selected as the research object and labeled as R1–3. The experiments were carried out using a phone camera to take snapshots of scrap to construct the base image. In past benchmarks, the GT of 3D information of real objects was obtained through laser scanner or LiDAR [15]. The same methodology was used in this paper to obtain GT using a SIMSCAN 30 handheld laser scanner with a maximum resolution of 0.02 mm. For scale calibration, a globe with a diameter of 85 mm was included in the scene. In addition, the thickness of scrap was measured using a thickness gauge. Due to different cross-sectional shapes, the average thickness measurements of 5 randomly selected areas were used as the true value. The GT acquired by the laser scanner of the real dataset, along with some of the dataset images, is illustrated in Figure 2b.

### 2.2. Multi-View Dataset of Unoccluded and Non-Overlapping Multiple Scrap Scenarios

**Figure 3:**
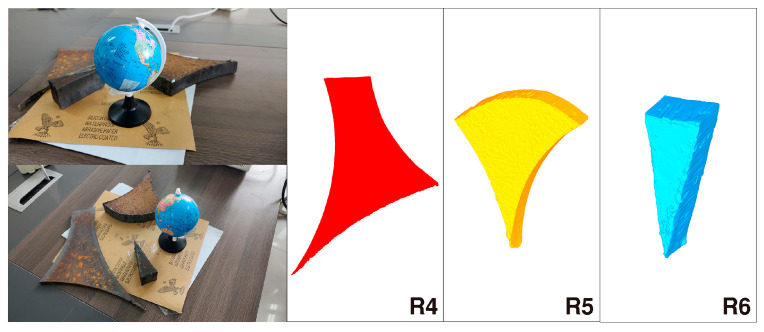
Multi-view dataset of multiple scrap under unoccluded and non-overlapping conditions.

A simple unobstructed scrap sorting and grading scenario was set up as a benchmark to study the automated processing flow based on 3D vision technology. The issue of occlusion in the scene requires specialized approaches to address it and will be investigated in future work. Three different scraps were placed in the scene as R4–6 with no accumulation, and then the dataset was processed according to the method described in Section 2.1. Representative sample images and corresponding GT point clouds for the multi-scrap metal scenario are presented in Figure 3.

Methodologically curated through physical capture and synthetic generation, the dataset comprises representative samples from three key scrap categories (industrial scrap, dismantling scrap, construction scrap), ensuring faithful representation of real-world scrap characteristics. To streamline the subsequent evaluation, this study utilizes scrap with relatively simple shapes rather than more complex varieties. To enable thickness quantification, geometrically simplified specimens are intentionally incorporated into the dataset architecture. The dataset maintains well-controlled illumination conditions with properly exposed scrap samples. While most real-world scrap-sorting workshops operate in indoor environments where industrial-grade lighting configurations can mitigate illumination variance, outdoor sorting facilities necessitate dedicated robustness evaluations for 3D systems under variable illumination, a critical research direction identified for subsequent investigation. Light changes can be addressed by moving operations indoors under controlled lighting or by using HDR technology. In contrast to 2D-based approaches plagued by persistent occlusion challenges, the multi-view imaging framework in our 3D solution inherently mitigates occlusion interference through viewpoint multiplicity. Table 2 summarizes the detailed information of the datasets.

## 3. Methods

The proposed pipeline comprises the following key steps: multi-view reconstruction, scale recovery, point cloud processing, and segmentation.

### 3.1. 3D Reconstruction Technology Based on Multi-View Reconstruction

Multi-view reconstruction, a technique for deriving quantitative measurements from photographic images, has emerged as a critical tool in industrial applications owing to its capacity to generate high-precision three-dimensional data in a non-destructive manner [16]. Key advantages are high spatial accuracy and resolution attainable under well-controlled conditions, non-contact measurement ideal for fragile or hazardous objects, cost efficiency by utilizing conventional imaging equipment, and the ability to acquire high-fidelity texture information [17]. In geotechnical engineering, smartphone-based photogrammetry effectively characterizes 3D geometric parameters of desiccation cracks, showing strong agreement with laser scanning (RMSE < 0.18 mm) despite limitations in occluded areas [16]. Similarly, in cryospheric studies, the time-lapse Structure-from-Motion (O-T-SfM 4D) method proves reliable for monitoring ice surface sublimation, with deviations generally below 0.09 mm/d compared to traditional weighing [18]. Both applications highlight the method’s advantages of low cost, efficiency, and field-deployable simplicity for quantitative 3D monitoring in challenging environments. Continued progress in computational algorithms and processing capabilities is further broadening its applicability in industrial automation and intelligent recognition systems. The subsequent section then reverts to discussing the core principles of multi-view geometry.

#### 3.1.1. Pixel to 3D Point: Monocular Camera Model

**Figure 4:**
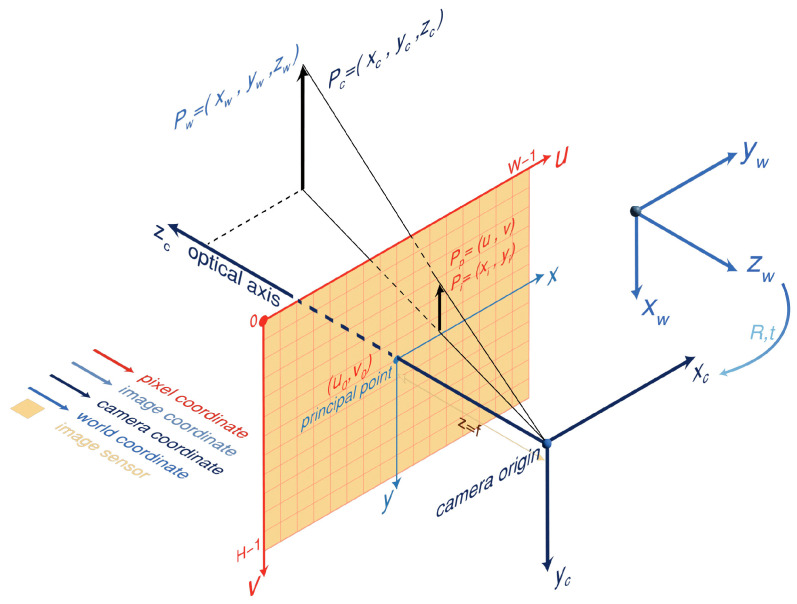
Schematic diagram of pinhole camera model.

The camera’s projection of 3D world points onto a 2D image plane can be described by a geometric model. The pinhole camera model is one of the relatively simple and effective methods of this study. As shown in Figure 4, the model involves four important coordinate systems of a pixel coordinate system, an image coordinate system, a camera coordinate system, and a world coordinate system. The pixel coordinate system is described as UV, with its origin at the top-left pixel of the image and the U and V axes along the sides of rectangular image. The coordinates in the pixel coordinate system have no physical units and only represent the indexes of the rows and columns of pixels. The image coordinate system describes the real coordinates of the imaging region in plane, with the origin at the center of imaging plane (called the principal point) and the *x* and *y* axes parallel to the *u* and *v* axes of the image pixel coordinate system, usually in mm. Equation (1) shows the relationship between the pixel coordinate system and the image coordinate system. The camera coordinate system takes the optical center of the camera as the origin of the coordinate system. *x_c_* and *y_c_* are parallel to the *x* and *y* axes of the image coordinate system, and the optical axis of the camera is the *z_c_* axis. The 3D coordinates of a point can be estimated from its 2D projections in multiple images based on the principle of triangulation, as expressed in Equation (2). The world coordinate system is used to represent the absolute coordinates of objects in space. The transformation from the camera coordinate system to the world coordinate system is demonstrated in Equation (3). Integrating Equations (1)–(3), the transformation from pixels to 3D points in a picture can be realized. The core of the transformation process is the two parts of intrinsic and extrinsic matrices. The intrinsic matrix remains constant once camera production is complete and can be obtained using Zhang’s calibration method [19], whereas the extrinsic matrix is typically derived from the relative motion of the camera. The transformation relationship between the pixel coordinates and the camera coordinate system in the normal coordinate mode can be obtained by combining Equations (1) and (2), as presented in Equation (5).

(1) $$\bar{P_{p}}=\left[\begin{array}{ccc} \frac{1}{dx} & 0 & u_{0}\\ 0 & \frac{1}{dy} & v_{0}\\ 0 & 0 & 1 \end{array}\right]\cdot \bar{P_{i}}$$

(2) $$z_{c}\cdot \bar{P_{i}}=\left[\begin{array}{cccc} f_{x} & 0 & 0 & 0\\ 0 & f_{y} & 0 & 0\\ 0 & 0 & 1 & 0 \end{array}\right]\cdot \bar{P_{c}}$$

(3) $$\bar{P_{c}}=\left[\begin{array}{cc} R & t\\ 0 & 1 \end{array}\right]\cdot \bar{P_{w}}$$

(4) $$\begin{array}{l} z_{c}\cdot \bar{P_{p}}=\left[\begin{array}{ccc} \frac{1}{dx} & 0 & u_{0}\\ 0 & \frac{1}{dy} & v_{0}\\ 0 & 0 & 1 \end{array}\right]\cdot \left[\begin{array}{cccc} f_{x} & 0 & 0 & 0\\ 0 & f_{y} & 0 & 0\\ 0 & 0 & 1 & 0 \end{array}\right]\cdot \left[\begin{array}{cc} R & t\\ 0 & 1 \end{array}\right]\cdot \bar{P_{w}}\\ =\left[\begin{array}{cccc} \frac{f_{x}}{dx} & 0 & u_{0} & 0\\ 0 & \frac{f_{y}}{dy} & v_{0} & 0\\ 0 & 0 & 1 & 0 \end{array}\right]\cdot \left[\begin{array}{cc} R & t\\ 0 & 1 \end{array}\right]\cdot \bar{P_{w}}=K_{3\times 4}\cdot T\cdot \bar{P_{w}}=K_{3\times 4}\cdot \bar{P_{c}} \end{array}$$

(5) $$z_{c}\cdot P_{p}=\left[\begin{array}{ccc} \frac{f_{x}}{dx} & 0 & u_{0}\\ 0 & \frac{f_{y}}{dy} & v_{0}\\ 0 & 0 & 1 \end{array}\right]\cdot P_{c}=K_{3\times 3}\cdot P_{c}$$

where (*u*_0_,*v*_0_) is the position of the principal point in the pixel coordinate system; *dx* and *dy* represent the true length of a pixel in the imaging sensor in the *x* and *y* directions; *f_x_* and *f_y_* are the focal lengths on the *x* and *y* axes, respectively; *R* is the rotation matrix and *t* is the translation matrix; *K*_3×4_ represents the camera intrinsic matrix, given by $\left[\begin{array}{cccc} \frac{f_{x}}{dx} & 0 & u_{0} & 0\\ 0 & \frac{f_{y}}{dy} & v_{0} & 0\\ 0 & 0 & 1 & 0 \end{array}\right]$; and *T* is the extrinsic matrix equal to $\left[\begin{array}{cc} R & t\\ 0 & 1 \end{array}\right]$. The horizontal line above the letter represents the form of homogeneous coordinates.

It is generally impossible to recover the structure of the entire 3D world from just one image. The following describes the geometry in a multi-view system.

#### 3.1.2. Camera Motion: Epipolar Geometry

**Figure 5:**
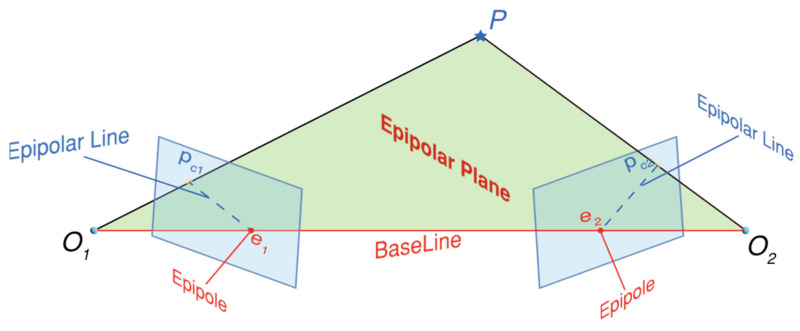
Schematic diagram of epipolar geometry.

Epipolar geometry is the geometric constraint between two perspective models, primarily for enabling binocular stereo vision and depth estimation via triangulation. In Figure 5, there are two cameras photographing an object *P* from different locations, with the centers of the cameras set to *O*_1_ and *O*_2_. The plane of observation, called *O*_1_*O*_2_*P*, is the epipolar plane. The intersections of the epipolar plane with the two image planes form the epipolar lines, labeled as *P_c_*_1_*e*_1_ and *P_c_*_2_*e*_2_. Epipole *e*_1_ is the projection of the right camera center *O*_1_ onto the left image plane, and likewise, epipole *e*_2_ is the projection of the left camera center *O*_2_ onto the right image plane [20].

According to Equation (6), the transformation from camera coordinate system 1 to camera coordinate system 2 can be realized.

(6) $$R_{12}P_{c1}+T_{12}=P_{c2}$$

*P_c_*_1_ and *P_c_*_2_ represent the observation point *P* in the camera coordinate system 1 and 2.

The cross product of *T* and the dot product of $P_{c2}^{T}$ on the left yield Equations (7) and (8).

(7) $$T_{12}\times R_{12}P_{c1}+T_{12}\times T_{12}=T_{12}\times R_{12}P_{c1}=T_{12}\times P_{c2}$$

(8) $$P_{c2}^{T}(T_{12}\times R_{12}P_{c1})=P_{c2}^{T}(T_{12}\times P_{c2})$$

Since *T* × *P_c_*_2_ represents the normal of the epipolar plane and is orthogonal to the vector *O*_2_*P*, $P_{c2}^{T}(T_{12}\times P_{c2})$ equals 0. Moreover, $P_{c2}^{T}(T_{12}\times R_{12}P_{c1})$ can be converted to Pc2TT12^R12Pc1 based on the property of the antisymmetric matrix. The transformation from Equation (8) to Equation (9) can be achieved.

(9) Pc2TT12^R12Pc1=0

By defining E=T12^R12, we can show the equivalence to Equation (10).

(10) $$P_{c2}^{T}EP_{c1}=0$$

Here, T12^ is the antisymmetric matrix of s$T_{12}$, and *E* is the essential matrix that constrains the spatial position relationship between the two perspectives.

Substituting Equation (5) and using the properties of the matrix transpose yields Equation (11).

(11) $$P_{p2}^{T}K^{-1^{T}}EK^{-1}P_{p1}=0$$

The matrix relation $F=K^{-1^{T}}EK^{-1}$ yields results equivalent to those in Equation (12).

(12) $$P_{p2}^{T}FP_{p1}=0$$

Here, F represents the fundamental matrix relating corresponding points in stereo images [21]. Typically, when *P_p_*_2_ and *P_p_*_1_ are known, the fundamental matrix F representing the camera motion relation can be solved using methods such as the eight-point algorithm [22], RANSAC [23], LMEDS [24], and so on. A scale factor ambiguity arises in the solution of Equation (12). Once *F* is available, together with the intrinsic matrix, *E* can be obtained. Finally, using the SVD decomposition method, *R*_12_ and *T*_12_ can be calculated from *E*.

#### 3.1.3. Depth Calculation: Triangulation

The relevant information on camera pose was obtained in Section 3.1.2, but still, the coordinates of the 3D points were not directly derived. Integrating Equations (5) and (6) yields Equation (13).

(13) $$R_{12}z_{c1}K^{-1}P_{p1}+T_{12}=z_{c2}K^{-1}P_{p2}$$

Defining ${\overset{⏜}{P}}_{c1}=K^{-1}P_{p1}$ and ${\overset{⏜}{P}}_{c2}=K^{-1}P_{p2}$ yields equivalence with Equation (14).

(14) $$z_{c1}R_{12}{\overset{⏜}{P}}_{c1}+T_{12}=z_{c2}{\overset{⏜}{P}}_{c2}$$

${\overset{⏜}{P}}_{c1}$ and ${\overset{⏜}{P}}_{c2}$ are termed normalized coordinates and serve as available parameters. Left-multiplying both sides of the equation by ${\overset{⏜}{P}}_{c2}$ gives Equation (15). Equation (15) contains only one unknown variable $z_{c1}$. Solving the equation yields $z_{c1}$, and subsequently, $z_{c2}$ can be solved.

(15) zc1P⏜c2^R12P⏜c1+P⏜c2^T12=zc2P⏜c2^P⏜c2=0

#### 3.1.4. Pixel Tracks: Point Correspondences Across Multiple Images

It is computationally challenging to find the corresponding pixel in another image for each pixel in the image. A common approach is to detect salient points in the image to facilitate a better search in another image. These points often appear at the corners of an image, texture changes and other places. The method of finding these corners is generally called a detector. In addition, we need to describe the characteristics of these points, such as the main direction of color change, etc., and match the corner points using these descriptions. These characteristics are typically encoded into vectors known as feature descriptors. Finally, we compare the similarity of these feature descriptor vectors to achieve the matching of points, and the method to implement this process is called a matcher. In recent years, many outstanding methods have emerged in this area due to the application of machine learning techniques. For example, there are FAST-ER, KeyNet, GLAMpoints, etc., for detectors, MKD, TFeat, etc., for descriptors, and SuperGlue, LightGlue, etc., for matchers. Some detector-free feature matching methods such as LoFTR have also been developed [25,26]. Nevertheless, from the perspective of the application scope, the traditional SIFT [27] algorithm is still the mainstream trusted algorithm. SIFT expands the scale-space information by Gaussian difference pyramids, allowing it to gain scale invariance and making it a landmark algorithm in the field of feature matching. For small inter-frame camera movements, correspondence searching via feature tracking (e.g., the KTL algorithm [28]) is computationally more efficient and thus better suited for real-time applications.

#### 3.1.5. Structure from Motion (SFM)

**Figure 6:**
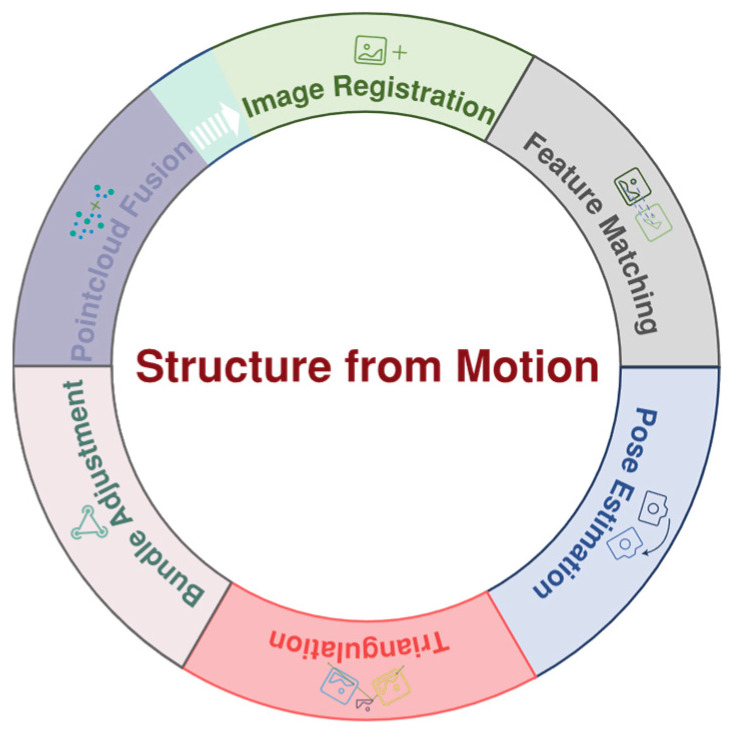
Schematic diagram of SFM.

The above sections focus on image information in two views. The core of the SFM algorithm, illustrated in Figure 6, involves a pipeline that extends the two-view methods discussed above to multiple images. A critical step within this pipeline is BA, solving the problem of error accumulation by jointly optimizing the positions of the 3D point cloud and the camera poses. Subsequently, the point cloud fusion process filters out existing spatial outliers and integrates new points from additional views to produce a consistent and complete 3D model.

#### 3.1.6. Multi-View Stereo (MVS)

SFM calculates the sparse point cloud and camera pose information of the scene; however, the sparse point cloud inherently represents only a partial reconstruction of the 3D scene information, and there is still a lot of 2D pixel information that is not used. The core objective of the MVS process is to estimate a dense 3D representation of a scene from a set of input images, leveraging known camera parameters (often from SFM) and under constraints of photometric consistency. For the feature points mentioned in Section 3.1.4, feature descriptors can be used to achieve pixel matching in multiple images, and searching for the position of non-feature points in another image is one of the core issues of MVS. If a corresponding relationship is found, the 3D point coordinates are calculated using the method described in Section 3.1.3. This process densifies the point cloud. According to the imaging principle, the color values of projected points from the same scene in two images are nearly identical. This property, known as photometric consistency, serves as one of the rules for pixel matching. However, the efficiency of traversing all pixels (2D search) of another image is low and the matching accuracy is also greatly reduced. In the case where the camera motion is given, the epipolar line is available, and the position of the matching pixel points must appear on the epipolar line from the two-view geometry. Searching for candidate pixels can be confined to the epipolar line, thereby reducing the problem to a 1D search, known as epipolar line searching. After the candidate pixels have been found, it is necessary to specify a metric to measure the color similarity between two pixels. However, comparisons based on single pixels are generally susceptible to noise and yield poor accuracy. Consequently, patch-based similarity comparisons are typically employed. Such a process is known as patch matching, and common algorithms include SSD, SAD, NCC, etc.

**Figure 7:**
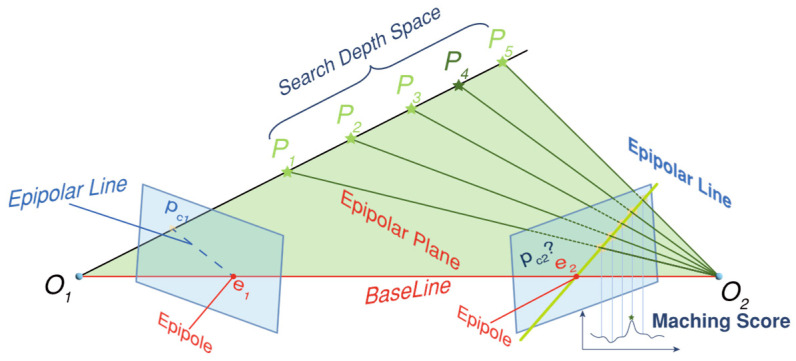
Schematic diagram of epipolar line search and patch matching.

A schematic diagram of epipolar line searching and patch matching is presented in Figure 7. In addition, due to the occlusion problem, some 3D points will not appear in all views, so visibility estimation is often performed to determine the specific perspective that needs to be evaluated for photometric consistency. More details can be found in these references [29,30,31].

### 3.2. Sphere Fitting Method to Solve the Uncertainty of Size Factor

3D reconstruction technology that relies solely on a monocular camera is unable to capture the true scale of an actual scene. In this study, a globe with a known diameter was placed in the scrap scene as a reference object for restoring the real scale factor. The selection of a sphere as a reference object is motivated by two main factors. Firstly, the globe exhibits a distinct, non-repetitive texture, enabling reliable detection and accurate matching during feature matching. Secondly, the distinct color contrast between the globe and the scrap, coupled with its spherical shape, makes it highly distinguishable, thereby simplifying the subsequent point cloud detection. The sphere fitting algorithm for point clouds is widely utilized in various fields, including part recognition, laser positioning, and robot calibration [32]. The spherical equation described by Equation (16) comprises four parameters, including the coordinates of the sphere’s center (*x*_0_, *y*_0_, *z*_0_) and its radius *r*_0_. From a mathematical point of view, four points are sufficient for sphere fitting. However, to enhance robustness, algorithms such as LM, RANSAC, or MSAC are often employed. The algorithm proceeds as follows. Four points are randomly selected to calculate the spherical equation. The distances from other points to the sphere are calculated. If the distance is less than (or equal to) a certain threshold, the point is considered an inlier. This process is repeated iteratively until the number of outliers is less than a preset value, and then iteration stops. Least squares spherical fitting is performed on the inliers to obtain the final spherical fitting coefficients. As a data structure enabling efficient storage and retrieval, KD-Tree is commonly used to organize point cloud data, facilitating rapid range and nearest-neighbor searches. Therefore, common point cloud processing frameworks such as PCL, Open3D, and Matlab(V2023B) all rely on KD-Tree algorithms as their foundation, and the sphere fitting algorithm is no exception. After obtaining the spherical parameters, the real size factor can be obtained using Equation (17). The coordinates of each point of the reconstructed point cloud are multiplied by the scale factor to recover the true scale of point cloud.

(16) $${\left(x-x_{0}\right)}^{2}+{\left(y-y_{0}\right)}^{2}+{\left(z-z_{0}\right)}^{2}=r_{0}^{2}$$

(17) $$RSF=\frac{D_{globe}}{2\cdot r_{fit}}$$

*RSF* represents the real size factor, $D_{globe}$ denotes the actual globe diameter of 8.5 cm, and $r_{fit}$ is the fitted spherical radius.

### 3.3. Point Cloud Data Processing and Segmentation

Point cloud data processing can be quite complex, often starting with the simplification of the point cloud through outlier-removal filtering algorithms in order to remove noise points and irrelevant data. Radius outlier removal is a common denoising technique based on the principle that each point, within a certain radius, must have a sufficient number of neighboring points to be retained, otherwise it is removed. Another method to reduce the computational load of point clouds is to use the pass-through filter to find ROI. Its working principle is to set a threshold range on the specified dimension of the point cloud and divide the data on this dimension into within the threshold range and not within the threshold range.

In practical industrial applications, scrap is always supported by a workbench. Consequently, the reconstructed point cloud contains not only the scrap object but also extensive point cloud data from the workbench surface, complicating the analysis of the scrap data. Since the workbench surface is generally a standard plane, it can be extracted from the point cloud using plane fitting methods. Similar to spherical fitting, the RANSAC method can also solve the plane equation, requiring only three points. In the iterative process of the algorithm, only three points are randomly selected to calculate the plane equation, as shown in Equation (18).

(18) ax+by+cz+d=0

When there are multiple scrap items or objects in the point cloud scene, we need to process them in blocks. This task is a point cloud segmentation problem, dividing the point cloud by spatial, geometric, and texture features to ensure that points in the same segment share similar features [33]. Among the segmentation algorithms developed based on the location information of point clouds is mainly the Euclidean clustering algorithm, while with the development of machine learning, unsupervised learning clustering algorithms such as Kmeans and DBSCAN have been applied in the task of point cloud segmentation. In addition, the Region Grow algorithm based on point cloud information is also a very effective segmentation algorithm. Representative deep learning algorithms for point cloud segmentation, such as PointNet++ [34] and Point-NN [35], exemplify the rapid development in this field.

## 4. Experiment and Analysis

### 4.1. Reliability: Accuracy Evaluation of Multi-View 3D Reconstruction Technology in Single-Scrap Scene

To reveal the feasibility of the proposed system, the accuracy of the multi-view reconstruction technique in a simple single-scrap scene was verified. The multi-view 3D reconstruction was implemented using the widely adopted Colmap (v3.8) [29,36], while point cloud processing was handled by Matlab 2022a and CloudCompare 2.13 alpha. The utilized operating system was Windows 10, and the hardware setup consisted of an AMD Ryzen 7 5800H CPU coupled with an NVIDIA GeForce RTX 3060 Laptop GPU.

#### 4.1.1. Scrap Size Measurement Algorithm Flow of Single Scrap Scene

**Figure 8:**
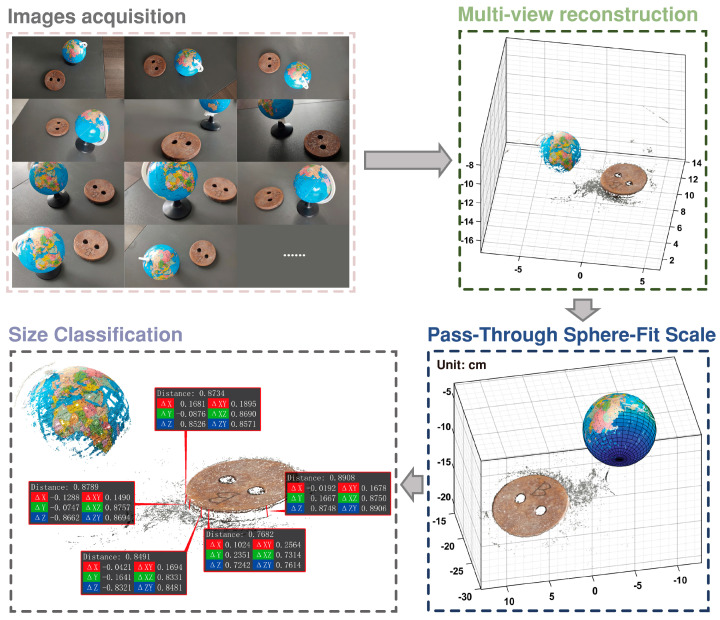
Schematic flow diagram of scrap size measurement for a single-scrap scene.

In this paper, each group of images was input into the Colmap system, and the default reconstruction parameters were retained by default for dense reconstruction. Next, the point cloud data was filtered by a pass-through filtering algorithm to obtain its ROI area. Then, through the MSAC algorithm, the spherical surface in the point cloud data was fitted, and the fitted sphere diameter was scaled to match the known true diameter of the calibration globe, and the size factor could be determined. Finally, the scrap thickness was measured by manually selecting points along the thickness dimension in the point cloud. The process is described in Figure 8.

#### 4.1.2. Metrics

In order to evaluate the results of multi-view reconstruction, we compared the reconstructed point cloud with GT provided by the dataset. Coarse registration was first performed by manually selecting corresponding points between the two point clouds followed by fine registration using the ICP algorithm [37]. Unlike the real dataset (with recovered scale), the synthetic dataset required scaling during alignment due to its unrecovered point cloud scale. To provide an unbiased evaluation of geometric accuracy [38], C2C distance calculation, accuracy and completeness metrics were applied.

C2C comparison refers to measuring the nearest-neighbor distance between corresponding points in two point clouds [39]. The C2C comparison process utilized the following criteria.

(19) $$RMSE=\sqrt{\frac{\sum_{i=1}^{N}X_{i}^{2}}{N}}$$

(20) $$MAE=\frac{\sum_{i=1}^{N}\left|X_{i}\right|}{N}$$

(21) $$STD=\sqrt{\frac{1}{N-1}\sum_{i=1}^{N}{\left(X_{i}-\bar{\bar{X}}\right)}^{2}}$$

where *N* denotes the number of observed point clouds, $X_{i}$ denotes the closest distance of each point to the corresponding reference point or surface, and $\bar{\bar{X}}$ denotes the average observed distance.

Accuracy and completeness, which are geometric metrics analogous to precision and recall under a distance threshold [39,40], involve measuring the distance between two point clouds. To assess accuracy, the distance from the computed data to the GT is calculated. Conversely, to assess completeness, the distance from the GT to the calculated data is calculated. Accuracy measures the alignment of reconstructed points with the ground truth, while completeness evaluates the coverage of all ground-truth points. A distance threshold is typically set to determine the percentage of points within an acceptable error margin.

#### 4.1.3. Results and Analysis on Multi-View Scrap Datasets

**Figure 9:**
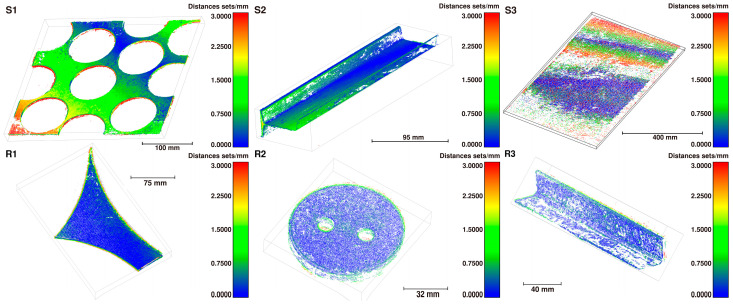
Color-coded C2C comparisons for multi-view scrap datasets.

C2C comparison. The point cloud distance was evaluated through C2C comparison based on Euclidean distance. The quantitative results, detailed in Figure 9 and Table 3, confirm that the multi-view reconstruction method yields sub-millimeter-level accuracy, with errors of <1.5 mm on synthetic datasets and <1 mm on real-world datasets, below the precision requirements in practical applications.

**Figure 10:**
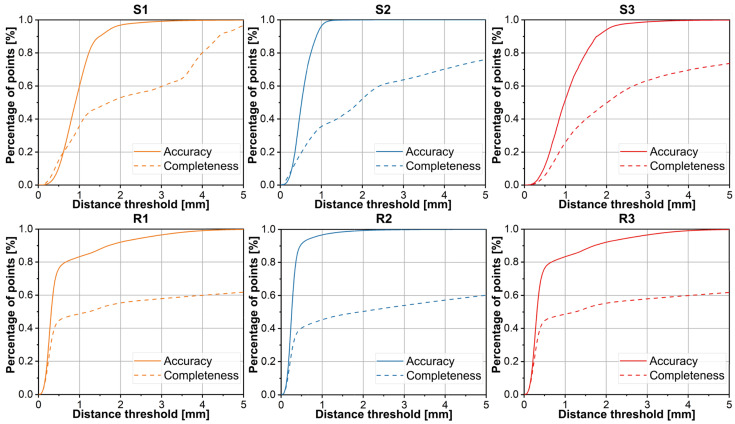
Accuracy and completeness on multi-view scrap datasets.

Accuracy and Completeness. The accuracy and completeness data are illustrated in Figure 10. The results reveal that the multi-view reconstruction achieves relatively high accuracy, though reconstruction completeness requires further improvement. As shown in Figure 9, insufficient lighting caused some scrap edges and faces to be unreconstructed, resulting in lost edge details that prevented correct feature matching.

Compared to the results on the synthetic dataset, those on the real dataset demonstrate superior performance. This can be attributed to the different scale recovery methods: the scale in the real dataset was recovered by fitting a calibration globe, whereas the ICP algorithm was used for the synthetic dataset. The ICP-based alignment may be less accurate when the completeness of the point cloud is low.

Thickness Comparison. As illustrated in Figure 8, the scrap thickness was manually measured using the point cloud data and compared against the actual thickness values. The results summarized in Table 4 show an absolute thickness error of approximately 0.5 mm, meeting the accuracy requirements for the scrap grading task.

### 4.2. Extensibility: A Pipeline for Scrap Grading Based on Multi-View 3D Reconstruction and Point Cloud Segmentation Technology

Section 4.1 demonstrates that the accuracy of the multi-view 3D reconstruction algorithm is fully applicable to the scrap grading task. We increased the complexity of the task from single-instance reconstruction to multiple scraps. Since the reconstruction output is point cloud data, it necessitates the use of point cloud processing techniques rather than relying on the more mature 2D image recognition technology. A fundamental approach involves segmenting the point cloud into individual scrap objects and then separately calculating the dimensional information for each segment. The segmentation step requires point cloud segmentation algorithms, while the sizing step can approximate the scrap dimensions by computing the minimum bounding box of each segment. Commonly used segmentation techniques are outlined in Section 3.3. The accuracy of the segmentation results directly determines the reliability of the subsequent dimensional measurements for individual scraps. This step represents a critical bottleneck that currently limits the application of 3D vision technology to large-capacity, industrial-scale grading and sorting scenarios. The study first evaluated the effectiveness of standard point cloud segmentation algorithms on a multi-view dataset featuring unoccluded and non-overlapping multiple scraps. Based on the evaluation, an automated grading pipeline for multi-scrap scenes, leveraging 3D vision, was then proposed.

#### 4.2.1. Effectiveness of Point Cloud Segmentation Algorithm on Multi-View Dataset of Unoccluded and Non-Overlapping Multi-Scrap Scene

IoU is an evaluation metric that quantifies the degree of overlap between two regions and is widely used in tasks such as segmentation and object detection. In the context of point cloud segmentation, IoU is calculated as the ratio of the number of points in the intersection to the number of points in the union of the predicted point set and the ground-truth point set. The mIoU is defined as the average of the IoU values across all categories. Furthermore, the running time of an algorithm is commonly employed to assess its execution efficiency.

**Figure 11:**
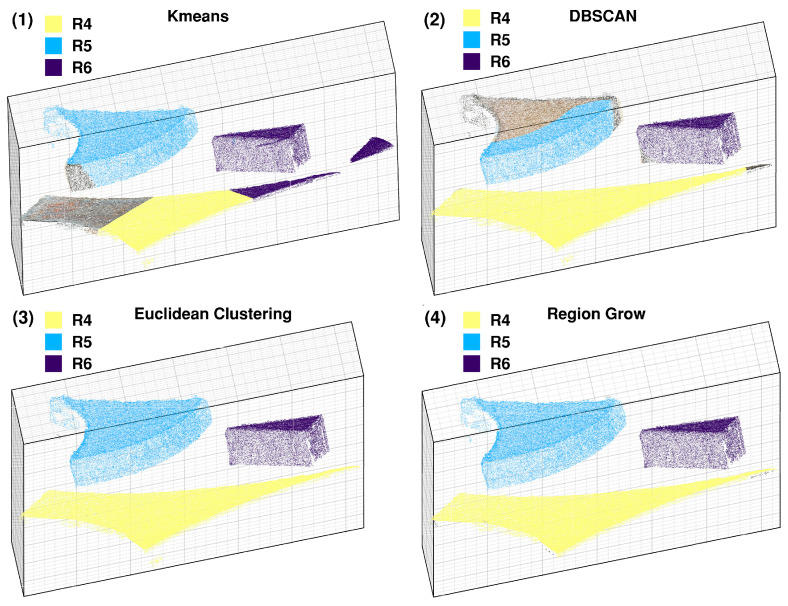
Schematic diagram of segmentation results.

As demonstrated in Figure 11 and consistent with the data in Table 5, both the Euclidean clustering and Region Grow algorithms achieve more precise boundary delineation. Furthermore, Table 5 verifies that the Euclidean clustering algorithm offers a significant computational advantage, being 11.3 times faster than the Region Grow algorithm. In contrast, the unsupervised-learning-based algorithms (K-means and DBSCAN) did not show a distinct advantage in this scenario. Considering both accuracy and efficiency, the Euclidean clustering algorithm yields the best overall results for processing point clouds in unoccluded and non-overlapping scrap scenes.

#### 4.2.2. An Automated Pipeline for Scrap Grading in Unoccluded and Non-Overlapping Scene Based on 3D Vision Technology

**Figure 12:**
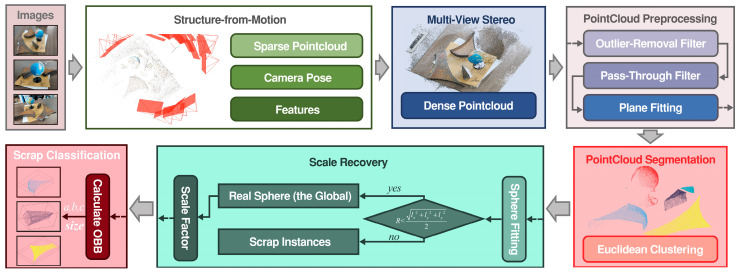
A pipeline for scrap grading in unoccluded and non-overlapping scene based on multi-view reconstruction and point cloud segmentation.

Building upon the processing pipeline for single scrap scenes in Section 4.1.1 and leveraging the point cloud segmentation effectiveness demonstrated in Section 4.2.1, this study proposes a technical workflow for multi-scrap grading scenarios, as outlined in Figure 12. A key operational insight is that attempting a direct spherical fit within a complex multi-scrap scene typically fails to accurately reconstruct the calibration globe. To address this, the optimized workflow prioritizes an initial point cloud segmentation step. Subsequently, spherical fitting is applied individually to each segmented point cloud cluster. An assessment of the results of automated processes was performed, as follows.

**Figure 13:**
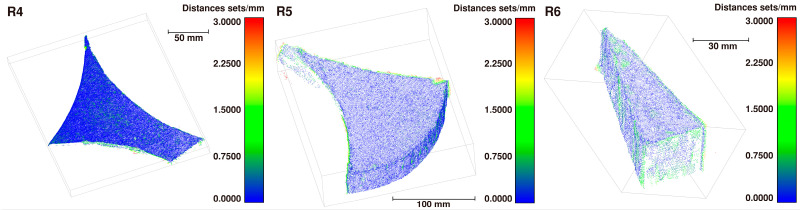
Color-coded C2C comparisons for multi-view dataset of unoccluded and non-overlapping multi-scrap scene.

C2C comparison. The detailed results of C2C comparison for the multi-scrap scene datasets are presented in Figure 13 and summarized in Table 6. Notably, the system maintains high reconstruction accuracy even in these more complex multi-scrap scenarios. This performance can be attributed to the robustness of the multi-view reconstruction pipeline and the effectiveness of the scale recovery method.

**Figure 14:**
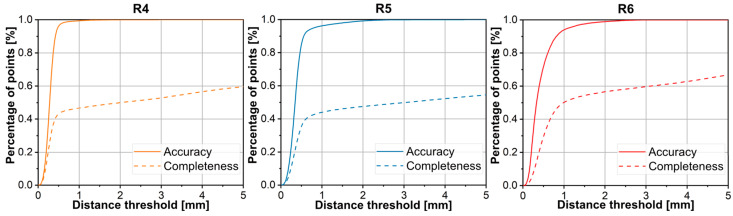
Accuracy and completeness on multi-view dataset of unoccluded and non-overlapping multi-scrap scene.

Accuracy and Completeness. Figure 14 presents the details regarding accuracy and completeness. The completeness of the point cloud remains low. In addition to the loss of edge and facial information, the surface of the scrap adjacent to the table side often cannot be reconstructed. This is significant because this surface typically occupies a large portion of the total area, contributing to the low overall completeness. However, this particular face can be recovered by fitting a plane equation, and it has a negligible impact on the dimensional measurements of plate-like scrap. Consequently, the completeness of the scrap sides and the preservation of edge point cloud information are more critical.

Thickness Comparison. The error in the thickness values is demonstrated in Table 7. The thickness detection error in the multi-scrap scene was less than 1 mm.

## 5. Industrial Application Outlooks

In this work, the grading process of scrap based on 3D vision technology is proposed. (1) For the scrap grading task, its purpose is mainly to further judge the value of scrap, and there is no need for further refined sorting, so there is no high requirement for the real-time performance of the algorithm’s flow. Simultaneously, during the processing of scrap, electromagnetic suckers are often used to transfer it. The flow of scrap materials is intermittent. Images are collected during this process, and the pipeline for automatic scene reconstruction and grading algorithms is executed in the background. The grading results are submitted to the production management system within minutes of completion. This workflow is considered to be reasonable, efficient, and inexpensive. (2) For the scrap sorting task, this process often works with mobile production lines and robotic arms. The efficiency of the algorithm based on multi-view reconstruction needs to be further improved. The multi-view reconstruction process builds a scene map, enabling robots or other mechanical structures to perform navigation and positioning.

## 6. Conclusions and Future Work

This work focuses on the many types of scrap, the complexity of actual recognition scenarios, and the difficulty of manual system interfacing, etc., and changing the current method of determining the scrap grade, which is mainly used by quality management personnel for visual recognition, into an intelligent grading method in most iron and steel enterprises. In order to solve the current generation of 2D vision-based scrap recognition technology, in which there are problems of non-interpretable 3D information inferred from 2D and poor robustness, a 3D vision-based scrap grading process is proposed, and the whole process is fully interpretable, while the accuracy meets the needs of scrap grading. Facing the task of scrap grading in scenes with unoccluded and non-overlapping multiple scrap items, an automated scrap detection pipeline based on multi-view reconstruction and point cloud segmentation technology is developed. The reconstruction accuracy, the effect of point cloud object segmentation, and the thickness error all meet the scrap classification standards.

Experiments were carried out on a single-scrap synthetic dataset, a single-scrap real dataset and a multi-scrap real dataset. The results show that the accuracy of the point cloud generated based on the multi-view reconstruction algorithm in the single-scrap scene was less than 1.5 mm, while the error of the manually measured scrap thickness based on the point cloud information was less than 1 mm. For the unoccluded and non-overlapping multi-scrap scenario, the proposed multi-view reconstruction fused with Euclidean clustering segmentation of the scrap detection process had an mIoU of 99.35%, and the segmentation processing time was 0.7430 s. The error in the plate scrap thickness measured using the OBB algorithm was also below 1 mm, meeting the accuracy required by the current national standard for scrap grading.

The complexity of 3D solutions remains challenging, primarily due to the high computational complexity of reconstruction algorithms and 3D recognition models. In order to promote the implementation of the next generation of scrap grading systems, the following problems remain to be solved in future research. (1) The multi-view reconstruction algorithm should be further optimized by switching to a more effective algorithm based on deep learning algorithms for feature matching, as well as configuring better lighting conditions and preserving dark details using HDR technology during the image acquisition process. (2) More efficient 3D reconstruction means, such as structured light cameras, LiDAR, etc., should be researched to realize the reconstruction of scrap scenes. (3) A more effective point cloud classification and segmentation algorithm based on deep learning should be proposed to realize the detection of scrap in accumulation scenes. (4) An automatic thickness measurement method for various shapes of scrap should be obtained. (5) Combined with modern spectroscopy technology, an online sorting process with complete scrap grading will be developed.

## Figures and Tables

**Table 1:** Criteria for classification of scrap (GB/T 39733-2024).

Types	Abbreviation	Dimension Requirements	Weight Requirements
Heavy recycling iron–steel materials	HRS	Thickness ≥ 6 mm or Diameter ≥ 10 mm Length ≤ 1500 mm Width ≤ 600 mm	Single weight ≤ 1500 kg
Medium recycling iron–steel materials	MRS	Thickness ≥4 mm or Diameter ≥8 mm Length ≤ 1500 mm Width ≤ 600 mm	Single weight ≤ 1500 kg
Light recycling iron–steel materials	LRS	Thickness ≥ 2 mm Length ≤ 1500 mm Width ≤ 600 mm	Single weight ≤ 1500 kg
Shredded recycling iron–steel materials	SRS	Packing density ≥ 0.8 t/m^3^	—
Bundled recycling iron–steel materials	BRS	Length ≤ 1500 mm Width ≤ 1000 mm Height≤ 1000 mm	Single weight ≤ 2000 kg
Alloy recycling iron–steel materials	ARS	Length ≤ 1500 mm Width ≤ 1000 mm	Single weight ≤ 1500 kg
Cast recycling iron–steel materials	CRS	Thickness ≥ 2 mm Length ≤ 1500 mm Width ≤ 600 mm	Single weight ≤ 1500 kg

**Table 2:** Detailed information of the datasets.

Scene	Single						Multiple
Type	Synthetic			Real			Real
Object	S1	S2	S3	R1	R2	R3	R4, R5, R6
Numb. images	20	20	40	27	19	20	25
Resolution	1920 × 1080			4000 × 2250			4000 × 2250
Ground truth	CAD model			Laser scanner			Laser scanner
Characteristics	Industrial	Construction	Dismantling	Dismantling	Industrial	Construction	Plate

**Table 3:** Metrics of C2C comparisons for multi-view scrap datasets (unit: mm).

Scrap	RMSE	MAE	STD
S1	1.11	0.98	**0.54**
S2	0.60	0.55	0.24
S3	**1.34**	**1.24**	0.51
R1	**1.06**	**0.63**	**0.85**
R2	0.44	0.32	0.30
R3	0.55	0.41	0.37

**Table 4:** Comparison of scrap thickness measurements with real values based on multi-view reconstruction algorithms (unit: mm).

Scrap	Real	Measured	Absolute Error
R1	5.82	5.40	0.42
R2	8.04	8.52	0.48
R3	2.34	2.30	0.04

**Table 5:** Evaluation of segmentation algorithms.

Algorithm	IoU			mIoU	Time/s	Platform
	R4	R5	R6			Windows10 CPU: Intel Core i5-8250
Kmeans	0.6112	0.9550	0.4764	0.6809	0.8319	Python 3.8.18 scikit-learn 1.3.2
DBSCAN	0.9889	0.5900	0.9819	0.8536	120.8351	Python 3.8.18 open3d 0.17.0
Euclidean clustering	0.9815	0.9993	0.9998	0.9935	0.7430	Matlab 2022b
Region Grow	0.9987	0.9996	0.9996	0.9993	8.2530	MSVC++ 14.3 PCL 1.13.0

**Table 6:** Metrics of C2C comparisons for multi-view dataset of unoccluded and non-overlapping multi-scrap scene (unit: mm).

Scrap	RMSE	MAE	STD
R4	0.33	0.29	0.16
R5	0.54	0.40	0.35
R6	**0.65**	**0.45**	**0.47**

**Table 7:** Comparison of scrap thickness measurements with real values based on multi-view dataset of unoccluded and non-overlapping multi-scrap scene (unit: mm).

Scrap	Real	Measured	Absolute Error
R4	5.82	6.55	**0.73**
R5	29.80	30.52	0.72
R6	36.00	35.55	0.45

## Data Availability

The data presented in this study are available on request from the corresponding author. The data are not publicly available due to an ongoing study.

## Abbreviations

The following abbreviations are used in this manuscript:

3D	Three-dimensional
DBSCAN	Density-based spatial clustering of applications with noise
Region Grow	Region growing segmentation
mIoU	Mean intersection over union
CV	Computer vision
CAD	Computer-aided design
GT	Ground truth
HDR	High dynamic range
RANSAC	Random sample consensus
LMEDS	Least median of squares
SVD	Singular value decomposition
1D	One-dimensional
SSD	Sum of squared differences
SAD	Sum of absolute differences
NCC	Normalized cross-correlation
LM	Levenberg–Marquardt
MSAC	M-estimator sample consensus
KD-Tree	K-dimensional tree
ROI	Region of interest
ICP	Iterative closest point
C2C	Cloud-to-cloud
IoU	Intersection over union
OBB	Oriented bounding box
LiDAR	Light detection and ranging
LRS	Light recycling iron–steel materials
MRS	Medium recycling iron–steel materials
HRS	Heavy recycling iron–steel materials
BA	Bundle adjustment
